# Postural Sway Velocity of Deaf Children with and without Vestibular Dysfunction

**DOI:** 10.3390/s24123888

**Published:** 2024-06-15

**Authors:** Renato S. Melo, Andrea Lemos, Carine Carolina Wiesiolek, Lucas Gallindo Martins Soares, Maria Cristina Falcão Raposo, Daniel Lambertz, Rosalie Barreto Belian, Karla Mônica Ferraz

**Affiliations:** 1 Department of Physical Therapy, Universidade Federal de Pernambuco (UFPE), Recife 50670-901, Brazilkarla.ferraz@ufpe.br (K.M.F.); 2 Post-Graduate Program in Physical Therapy, Universidade Federal de Pernambuco (UFPE), Recife 50670-901, Brazil; 3 Laboratory of Pediatric Studies (LEPed), Universidade Federal de Pernambuco (UFPE), Recife 50670-901, Brazil; 4 Post-Graduate Program on Child and Adolescent Health, Universidade Federal de Pernambuco (UFPE), Recife 50670-901, Brazil; 5 Laboratory of Informatics in Health, Institute Keizo Asami (iLIKA), Recife 50670-901, Brazil; 6 Department of Statistics, Universidade Federal de Pernambuco (UFPE), Recife 50670-901, Brazil; 7 Department of Medicine, Universidade Federal de Pernambuco (UFPE), Recife 50670-901, Brazil

**Keywords:** child, cochlear implant, deafness, degrees of the hearing loss, hearing impairment, motor skills disorders, postural balance, postural control, stability, vestibular diseases

## Abstract

Background: Sensory information obtained from the visual, somatosensory, and vestibular systems is responsible for regulating postural control, and if damage occurs in one or more of these sensory systems, postural control may be altered. Objective: To evaluate and compare the postural sway velocity between children with normal hearing and with sensorineural hearing loss (SNHL), matched by sex and age group, and to compare the postural sway velocity between children with normal hearing and with SNHL, with and without vestibular dysfunction. Methods: Cross-sectional study that evaluated 130 children (65 with normal hearing and 65 with SNHL), of both sexes and aged between 7 and 11 years, from public schools of the city of Caruaru, Pernambuco state, Brazil. The postural sway velocity of the center of pressure (COP) was assessed by a force platform, in two directions, anteroposterior (AP) and mediolateral (ML)), in three positions, namely bipedal support with feet together and parallel (parallel feet (PF)), bipedal support with one foot in front of the other (tandem foot (TF)), and single-leg support (one foot (OF)), evaluated with the eyes open and closed. Results: Children with SNHL demonstrated greater postural sway velocity compared to children with normal hearing in all the positions evaluated, with significant differences in the AP direction, with the eyes open (PF: *p* = 0.001; TF: *p* = 0.000; OF: *p* = 0.003) and closed (PF: *p* = 0.050; TF: *p* = 0.005). The same occurred in the ML direction, with the eyes open (PF: *p* = 0.001; TF: *p* = 0.000; OF: *p* = 0.001) and closed (PF: *p* = 0.002; TF: *p* = 0.000). The same occurred in relation to vestibular function, where the children with SNHL with an associated vestibular dysfunction demonstrated greater postural sway velocity compared to children with normal hearing in all the positions evaluated, demonstrating significant differences in the AP direction, with the eyes open (TF: *p* = 0.001; OF: *p* = 0.029) and eyes closed (PF: *p* = 0.036; TF: *p* = 0.033). The same occurred in the ML direction, with the eyes open (TF: *p* = 0.000) and with the eyes closed (PF: *p* = 0.008; TF: *p* = 0.009). Conclusions: Children with SNHL demonstrated greater instability of postural control than children with normal hearing in all the directions assessed. Children with SNHL and an associated vestibular dysfunction demonstrated the greatest instability of postural control in this study.

## 1. Introduction

Postural control is the process of maintaining the center of pressure (COP) by projecting the center of gravity onto the ground, which occurs within the base support of the feet and requires continuous postural adjustments of neuromuscular activity and joint positioning to achieve and/or maintain balance stability [1,2,3]. Sensory information from the visual, somatosensory, and vestibular systems are responsible for regulating postural control, being integrated and selected by central processing, to generate adequate motor responses and thus control, coordinate, and maintain postural stability [4].

In children, the stability of postural control is essential for developing motor skills and is an essential prerequisite for most activities of daily living, recreation, and leisure [5]. It is the complex motor skill of maintaining, achieving, or restoring a state of body balance while the child is still, preparing to move, moving, or preparing to stop moving [6,7]. Thus, if there is a change in one or more of these sensory systems responsible for regulating postural control, the postural stability and balance of these children may be altered [8,9,10,11,12,13,14,15].

Peripheral vestibular dysfunctions have been a frequent finding in children and adolescents with sensorineural hearing loss (SNHL), identified in 41–89% of the samples from several studies [16,17,18,19,20,21]. This high prevalence of vestibular dysfunction in children with SNHL occurs due to the anatomical proximity between the cochlea and the vestibule, organs that share the continuous membranous labyrinth of the inner ear [22]. Thus, prenatal, perinatal, or postnatal injuries and/or trauma can cause damage in one or both systems, predisposing children with SNHL to present with peripheral vestibular dysfunctions, due to the injury in the inner ear [23,24,25,26].

Given these evidence, it is justified to evaluate the postural control of children with SNHL; however, some investigations have chosen to evaluate the postural control using clinical tests or scales [27,28,29], which may be less sensitive instruments for detecting minor magnitude changes in children with SNHL. Other authors have opted for computerized dynamic posturography and force platforms to analyze the postural control of children with SNHL [30,31,32]. However, despite being accurate instruments for this type of assessment in children [33,34,35,36], which includes children with SNHL [37], these studies did not perform calculations to estimate the sample size of their studies, making it difficult to generalize their results.

In addition to the limitations regarding the accuracy of the instruments, and the lack of calculations for the estimation of sample sizes, there is also a need to control specific characteristics of the children with SNHL which can influence postural control, such as degrees of hearing loss and vestibular function. Some studies have proposed to evaluate the postural control and motor disorders of children with SNHL according to the degree of hearing loss [38,39,40]; however, these studies did not evaluate the function of the vestibular system of the samples, and the vestibular function would be an important control measure in this case, as evidence shows that there is a relationship between the presence of vestibular dysfunction and greater degrees of hearing loss in children with SNHL [41,42,43,44,45].

Furthermore, there is still a lack of research in the literature that has evaluated the postural control of children with SNHL and correlated such findings with the vestibular function of these children. However, when previous investigations have made this correlation, the children’s postural control was measured by clinical tests and/or scales [46,47,48,49]. In view of the above, the assessment of the postural control in children with SNHL carried out using more sensitive and accurate methods to detect changes in the COP, associated with the assessment of the vestibular function, in a sample size properly estimated through sample calculation, is scarce in the current literature thus justifying the carrying out of this study.

Therefore, the primary objective of this study was to evaluate and compare the postural sway velocity between the children with normal hearing and those with SNHL, matched by sex and age group. The secondary objective was to compare the postural sway velocity of the children with SNHL, according to the degrees of hearing loss, and the function of the vestibular system with a sample of children with normal hearing.

## 2. Methods

### 2.1. Study Design and Sample

This study comprised a cross-sectional analysis of 130 children, divided into the following two groups: 65 children with normal auditory capabilities and 65 children diagnosed with SNHL. The participants consisted of both genders and were aged between 7 and 11 years, the age at which postural stability is already mature in the children [50]. The children were selected from two public educational institutions within the same municipality in Caruaru, Pernambuco state, Brazil. These schools share comparable socioeconomic environments, and one specifically caters to students with hearing impairments.

This study was evaluated and approved by the Research Ethics Committee of the Health Sciences Center of the Federal University of Pernambuco (CEP/UFPE), according to final protocol nº. CAAE: 0504.0.172.000-11, in accordance with resolution 466/12 of the National Health Council.

To compute the necessary sample size, a frequency occurrence of 87% for “static balance changes in children with SNHL” [51] was considered, with an acceptable margin of error set at 10%. The calculation was performed using the OpenEpi tool (www.openepi.com (accessed on 29 August 2023)) [52], which suggested a sample size of 123 children. To ensure demographic parity in age and gender, an additional 7 children were included, bringing the total to 130, with 65 in each group.

The criteria for inclusion applicable to both groups required that the children be enrolled at one of the two participating schools and aged between 7 and 11 years. For children with SNHL, the specific inclusion criteria were also set as follows: a confirmed clinical diagnosis of bilateral SNHL, completion of auditory and vestibular evaluations, and proficiency in Brazilian Sign Language to guarantee comprehension of instructions during the study. It is pertinent to mention that instructions were provided in Brazilian Sign Language at their respective schools.

The exclusion criteria for all the participants included any neurological, physical, visual, or mental impairments; a history of head and/or neck trauma; and a leg length discrepancy exceeding 2 cm as determined by prior assessments conducted by the examiner. For the children with normal hearing, additional criteria excluded those with any form of hearing impairment or experiences of dizziness. Eligibility for the study was determined through interviews with parents and teachers, analysis of school records, medical documentation, and a physical examination conducted by the examiner before the balance assessment.

Participation in this study was contingent upon obtaining parental or legal guardian consent through the signing of a free and informed consent form, which authorized the involvement of their children and the subsequent publication of the study’s findings. Recruitment of participants was conducted sequentially, with children being sex- and age-matched and selected through a randomized drawing process facilitated by their teachers, who were not informed about the specific objectives of the study.

This randomization process used two opaque envelopes, one containing the names of boys and the other containing the names of girls, organized by age group. To achieve sex and age matching as per the study’s design, teachers were instructed to randomly select 6 boys and 7 girls between the ages of 7 and 11. The selection of a greater number of girls was due to their higher enrollment numbers at the participating schools. These drawings took place in the classroom, witnessed by all the students and the research team.

The study procedures, particularly those related to the examination and assessment of static balance, were communicated verbally to the children with normal hearing. For children with SNHL, the explanations were provided by a researcher proficient in Brazilian Sign Language, ensuring that all the participants fully understood the procedures involved.

### 2.2. Assessment of the Postural Sway Velocity

The analysis of postural sway velocity was employed to evaluate the postural stability of the children, utilizing a force platform (Biomec 400, EMG System; São José dos Campos SP, Brazil). This device recorded the Center of Pressure (COP) sway velocity data in centimeters per second, assessing movements in both the anteroposterior and mediolateral directions. Measurements were taken in three different standing positions on the platform, both with eyes open and closed. These tests were conducted within a controlled environment in a quiet room, with only the examiner present, to minimize any external disturbances.

For this study, the force platform was set to operate with a sampling frequency of 100 Hz and an amplifier gain of ×600. Following each of the six assessment sessions, the platform’s software generated data on the COP sway velocity for each participant. It is important to note that an increased COP sway velocity indicates a greater delay in the compensatory postural adjustments which are necessary to stabilize the child’s posture [53,54,55,56,57].

### 2.3. Procedures

The dataset collected from the children encompassed demographic and physical details including identification, dominant hand, date of birth, height, and weight. The researchers outlined the procedures that would be undertaken during the assessment of static balance. This involved the collection of anthropometric data and stabilometric measurements as detailed subsequently.

**Figure 1:**
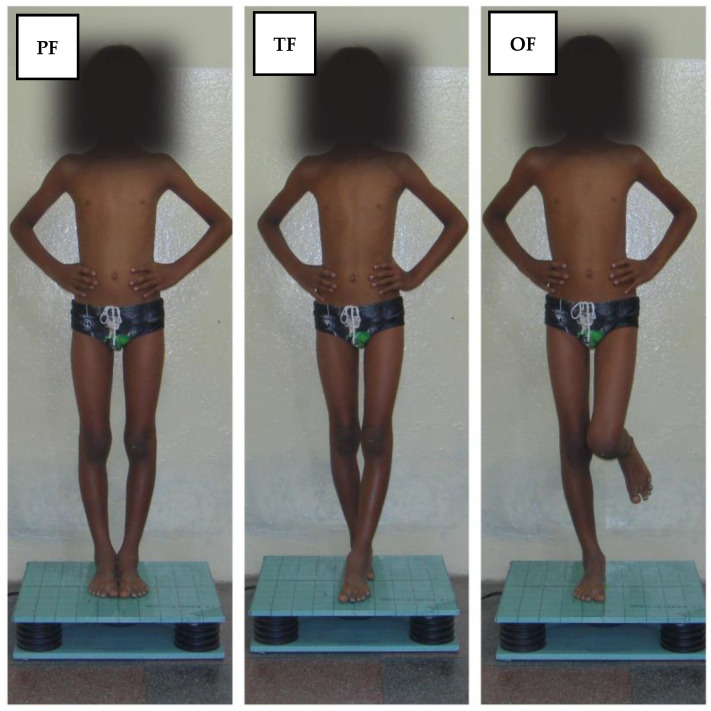
Positions in which each child was evaluated: PF: parallel feet; TF: tandem feet and OF: one foot.

For the acquisition of the COP sway velocity data, children were positioned at the center of the force platform, with their hands resting on their iliac crests. Their feet were placed in three distinct support bases as follows: bipedal support with feet together and parallel (PF), tandem feet (TF), and unipedal support on the dominant leg (1 foot, OF), as depicted in Figure 1. Each posture was maintained for 30 s with their eyes open, followed by a repetition of the series with their eyes closed, culminating in a total of six acquisitions on the force platform.

Following the stabilometric assessment, children diagnosed with SNHL underwent two further examinations as follows: audiometry, to evaluate the severity of the hearing loss, and computerized vector electronystagmography, to assess vestibular function.

Before these auditory and vestibular tests, caregivers were advised to withhold caffeine-containing beverages, as well as narcotic and antivertigo medications (under medical supervision), from the child’s intake for 72 h. This precaution was taken to mitigate any potential interference with the outcomes of the examinations, particularly the vestibular assessment.

### 2.4. Hearing Assessment

The hearing assessment was conducted by an audiologist with 14 years of specialization in pediatric hearing evaluations, utilizing an acoustic test booth equipped with an audiometer (Beta Medical 6000; São Paulo, SP, Brazil). The procedures executed included pure-tone audiometry at the frequencies of 1000, 2000, 3000, 4000, 6000, 8000, 500, and 250 Hz. Additionally, vocal audiometry was performed, utilizing the tritonal mean derived from the pure-tone audiometry results.

Hearing normality was determined based on thresholds up to 20 dB. To categorize the severity of Sensorineural Hearing Loss (SNHL), the criteria set forth by the British Society of Audiology were applied. According to these guidelines, SNHL is classified into the following four levels based on the decibel thresholds observed: mild (21–40 dB), moderate (41–70 dB), severe (71–95 dB), and profound (greater than 95 dB) [58].

### 2.5. Vestibular End-Organ Assessment

For the examination of the vestibular end-organs, precise placement of the electrodes on the child’s face was essential, including a ground electrode positioned in the superolateral region of the eyeball to capture horizontal eye movements and central monocular vision. Additional electrodes were attached infraorbitally and supraorbitally to detect the presence or absence of nystagmus.

The vestibular evaluation comprised three distinct tests, namely positional, rotational chair, and caloric tests. The positional examination involved the child seated and utilized a light-emitting diode light bar for various observations, including calibration, detection of spontaneous (with both eyes open and closed), and semi-spontaneous nystagmus (directionally to the right, left, upward, and downward), saccadic eye movements, pendular tracking, and optokinetic nystagmus.

In the rotational chair test, which focuses on assessing the horizontal semicircular canal, children were securely fastened in the chair with a seat belt within a completely darkened environment. They were instructed to maintain three head positions as follows: bent forward, extended, and rotated to both the right and left, with their eyes closed. In each of these positions, the chair was rotated alternately to the right and left, employing a sinusoidal harmonic acceleration stimulus at frequencies of 0.01, 0.05, and 0.1 Hz with a peak velocity of 50 degrees per second. The outputs, including parameter gain, phase, and asymmetry, were quantified using methodologies as described by Maes et al. [19] and Maes et al. [59] to provide normative data for the rotatory test in pediatric populations.

The caloric test was administered using a calorimeter (Neurograff-Eletromedicina; Santa Rita do Sapucaí, MG, Brazil), employing air as the medium. Conducted while the child was in a supine position, the test involved both warm (50 degrees) and cold (24 degrees) air stimulations, with additional colder stimulations (10 degrees) applied as necessary. This approach allowed for the observation of nystagmus laterality and the determination of the type of vestibular lesion, whether irritative or deficient.

Reference values for interpreting the caloric test results were adopted as follows: for unilateral vestibular hyporeflexia, the sum of the slow component angular velocity (SCAV) values from both the cold and hot tests on the right and left ears was less than 5 degrees per second; for bilateral, the sum was less than 12 degrees per second. For unilateral vestibular hyperreflexia, the sum of the SCAV in the cold and hot tests in the right and left ears exceeded 62 degrees per second, and for bilateral hyperreflexia, the sum in all four tests exceeded 122 degrees per second, according to criteria from Albertino et al. [60].

Subsequently, a detailed report on the vestibular function assessment was generated, classifying each child’s vestibular condition as either normal or indicative of vestibular dysfunction. Following the auditory and vestibular evaluations, the group of children with SNHL was stratified into four subgroups—two based on the degree of SNHL (mild and moderate, or severe and profound) and two based on vestibular function (normal or dysfunctional), assessed by the caloric and/or rotational tests.

Out of the 65 children with SNHL, 38 (58.5%) displayed a normal vestibular reflex, while 27 (41.5%) exhibited vestibular dysfunctions, leading to subgroup formation based on vestibular function. Among the 38 children with a normal vestibular reflex, 12 had mild or moderate SNHL, and 26 had severe or profound SNHL, further categorizing them based on SNHL severity.

It is crucial to note that among those with mild and moderate SNHL, two children used hearing aids, and five used cochlear implants. Considering the potential influence of these auditory devices on the results, analyses between the subgroups excluded these children. However, given the unresolved questions surrounding the impact of cochlear implants on the vestibular function and balance in children with SNHL, the results from the five children who used cochlear implants are presented descriptively and separately in this study.

### 2.6. Data Analysis

The data analysis encompassed a variety of statistical techniques and measures to assess the differences and relationships within the study parameters. Proportions, means, and standard deviations were utilized as descriptive measures, while the Kolmogorov–Smirnov test was applied to evaluate the normality of quantitative variables. For comparing the means between two groups with normally distributed data, Student’s t-test (preceded by the Levene test to assess the homogeneity of variances) was employed. Conversely, the Mann–Whitney test was used for non-normally distributed data. Qualitative variables were analyzed using Pearson’s chi-squared test.

Data were initially tabulated in Microsoft Excel and subsequently analyzed using the Statistical Package for the Social Sciences (SPSS). A significance level of 5% (*p* ≤ 0.05) was established for all analyses to determine statistical significance. To mitigate the risk of alpha error, nonparametric analyses such as the Kruskal–Wallis test were utilized to compare multiple groups, particularly assessing the differences between children with normal hearing and those with varying degrees of SNHL, and the differences based on vestibular function.

Specifically, comparisons were made among the three groups categorized by degrees of SNHL (children with normal hearing, mild/moderate SNHL, and severe/profound SNHL) and between the groups based on vestibular function (normal hearing, normal vestibular function, and vestibular dysfunction) across six evaluated positions. Significant differences (*p* = 0.000) were observed in most comparisons, with the exception of the sixth position (*p* = 0.028) in the SNHL degree comparison.

Post hoc comparisons were conducted between pairs of groups (e.g., normal hearing vs. mild/moderate SNHL, normal hearing vs. severe/profound SNHL, etc.) within each category (SNHL degrees and vestibular function). Holm–Bonferroni correction was applied to adjust the *p*-values for multiple comparisons, ensuring appropriate control of the family-wise error rate. This systematic approach allowed for rigorous statistical analysis and interpretation of the data, facilitating robust conclusions to be drawn from the study findings.

## 3. Results

The sample characterization data of this study are shown in Table 1. Children with SNHL showed greater postural sway velocity compared to children with normal hearing in all the positions evaluated, demonstrating significant differences for all the positions with eyes open and in the PF and TF positions with eyes closed, in the antero-posterior and medio-lateral directions, respectively, as shown in Table 2.

There were no significant differences between the postural sway velocity of the children with normal hearing and children with mild and moderate hearing loss in any of the positions evaluated, in the antero-posterior or medio-lateral directions. However, when comparing children with normal hearing and children with severe and profound hearing loss, children with hearing loss showed the highest postural sway velocity in all positions evaluated with eyes open, in the antero-posterior and medio-lateral directions, and in the PF in the medio-lateral direction and TF in both directions, with their eyes closed, as illustrated in Table 3 and Table 4.

When comparing the subgroups of children with SNHL, according to the degrees of hearing loss, there were no significant differences in any position evaluated in either direction. However, children with severe and profound SNHL showed greater postural sway velocity in almost all the positions compared to children with mild and moderate SNHL (Table 3 and Table 4).

When comparing the postural sway velocity of the children with SNHL, according to the vestibular function presented, it was observed that children with SNHL and normal vestibular function demonstrated greater postural sway velocity than children with normal hearing in all the positions evaluated with their eyes open, demonstrating significant differences in both directions. In the assessments with their eyes closed, the children with SNHL and normal vestibular function also demonstrated greater postural sway velocity, and significant differences were observed in PF in the medio-lateral direction and TF in both directions, according to Table 5 and Table 6.

In the comparison between children with normal hearing and those with SNHL and associated vestibular dysfunction, the postural sway velocity was greater in children with hearing loss, demonstrating significant differences in the TF and OF positions, in the antero-posterior direction and in the TF position in the medio-lateral direction, with open eyes. In the assessment with their eyes closed, significant differences were observed in the PF and TF positions, in both directions (antero-posterior and medio-lateral), with children with SNHL and vestibular dysfunction always showing greater postural sway velocity, as shown in Table 5 and Table 6.

There were no significant differences between the postural sway velocity of children with SNHL with and without vestibular dysfunction. However, children with SNHL and associated vestibular dysfunction showed the highest postural sway velocity in all positions evaluated and in both directions, as shown in Table 5 and Table 6.

Data on the means and standard deviations of the postural sway velocity of children undergoing cochlear implant surgery were presented, descriptively, in the antero-posterior and medio-lateral directions in Table 7.

Regarding the vestibular findings of this study, of the 27 children with SNHL and associated vestibular dysfunction, 25 (92.6%) exhibited severe and profound degrees and 2 (7.4%) exhibited mild and moderate degrees; moreover, 5 children (7.7%) in the sample with SNHL used cochlear implants, and all of them showed severe and profound degrees of SNHL and associated vestibular dysfunction.

Of the 27 children with vestibular dysfunction, 18 (66.7%) exhibited bilateral hyporeflexia, 6 (22.2%) exhibited unilateral hyporeflexia, and 3 (11.1%) exhibited bilateral hyperreflexia.

## 4. Discussion

Children with SNHL of this study demonstrated worse postural stability when compared to children with normal hearing. This worse postural stability was represented by a greater postural sway velocity and showed significant differences in all positions evaluated with eyes open, as well as in two positions with eyes closed, in both directions (antero-posterior and medio-lateral).

This worsened stability in postural control found in the group with SNHL may be related to the vestibular dysfunction found in this sample, which also corroborates other investigations, which have reported that children with SNHL often present vestibular dysfunctions [61,62,63]. When present, a vestibular dysfunction appears to alter important tonic–postural reflexes, such as the vestibulo-ocular and vestibulo-spinal reflexes, responsible for regulating head and postural stability, respectively. This would justify why the children with SNHL evaluated here had a higher postural sway velocity compared to children with normal hearing, also corroborating other investigations which observed similar results [64,65,66] that may or may not have used the same instruments as this study but had a smaller size sample.

Another interesting finding of this study is that the postural sway velocity of the children with SNHL increased as the assessments were carried out without visual cues, and when the width of the support base of these children’s feet was reduced. These findings corroborate other studies that also observed that children with SNHL use the visual and somatosensory systems to compensate their vestibular and balance deficits [67,68], thus this highlights the importance of the visual and somatosensory systems for regulating postural control in childhood.

Regarding the postural stability and the degrees of hearing loss, there were no significant differences between the postural sway velocity of the children with normal hearing and those with mild and moderate hearing loss in any position or direction evaluated.

This finding may be related to the reduced difficulty with hearing of the group with mild and moderate hearing loss, as evidence suggests that hearing can contribute by serving as fixed points of environmental reference [69,70,71,72]. In this way, such children, with greater hearing capacity, can still focus on these auditory points and obtain better spatial orientation and postural stability. In this sense, several investigations have reported that the postural stability of children with SNHL improved with the use of cochlear implants and individual sound amplification devices and justified that this improvement occurred due to the possibility of the hearing ability provided by these hearing devices [73,74,75,76,77,78].

These evidence can also explain why children with severe and profound hearing loss had the highest postural sway velocities when the outcome was analyzed in relation to the degrees of hearing loss. The greater difficulty these children had with hearing may have contributed to this as auditory information improves postural stability, reducing the postural sway in the antero-posterior and medio-lateral directions, and consequently serving as an underlying sensory system to reorganize postural stability and motor skills of children with SNHL [77,78,79].

These findings regarding to the degrees of hearing loss demonstrate that children with severe and profound hearing loss should have the priority to be evaluated and included in balance rehabilitation programs, not only due to the worst performances presented here, but also due to the possibility of presenting vestibular dysfunction concomitant to SNHL.

When the postural sway velocity was analyzed regarding to the function of the vestibular system of the children with SNHL, significant differences were identified in four positions evaluated in the antero-posterior direction and in five positions in the medio-lateral directions, with the children with SNHL and normal vestibular function always presenting with the highest postural sway velocity compared to their hearing peers.

This finding demonstrates that even with normal vestibular function, children with SNHL still present with disorders in postural stability, demonstrating, as mentioned above, that the ability to hear appears to contribute to the regulation and organization of postural stability in children. This finding corroborates recent investigations that observed that even in conductive hearing loss [80], and with the potential use of noise-blocking or noise-amplifying headphones, balance can be altered [81,82,83], auditory input is not neutral in the motor skills of children with SNHL, and it represents important sensory information when stimuli visual and somatosensory are modified [84,85].

These results suggest that auditory information plays an important role in children’s postural stability, and that isolated auditory changes may be capable of changing postural stability in this population, even when vestibular function is preserved, as observed in this study. Given this finding and the other evidence cited above, which demonstrated that auditory information appears to influence children’s postural stability, the following question arises: Would this be the ideal time to break paradigms and include auditory input as another piece of information sensory system responsible for regulating human postural stability [86]?

The importance of auditory input for postural stability can be reinforced and evidenced in the findings that show that in the presence of vestibular dysfunction associated with hearing loss, the greatest postural instabilities in all positions evaluated occurred as both systems (cochlear and vestibular) were altered in the children of this study.

These findings corroborate the results of other studies, which have evaluated the postural stability of children with hearing loss according to vestibular function and concluded that children with SNHL and associated vestibular dysfunction present the greatest postural instabilities in their samples. This occurred even though such studies had a smaller sample size and postural stability was assessed using instruments other than those in the present study [87,88,89,90,91]. The presence of vestibular dysfunction associated with hearing loss appears to be a predictor for worse performance in balance and postural stability, including differences in the patterns of postural oscillations in children with SNHL.

Adequate postural stability is necessary to perform most recreational and leisure activities in childhood, so the results found here may trigger the relevant motor disorders and harm the full development and motor performance of children with SNHL. In this sense, some changes have already been described in the literature, such as in motor skills [92,93,94,95] and gait [96,97,98,99], and reduced participation in school and sports activities of children with SNHL [100,101], such that these children are not comparable to children with normal hearing in terms of development and motor performance [102]. This can trigger emotional problems and depressive symptoms in children with SNHL [103,104], demonstrating the need to include these children in vestibular and balance rehabilitation programs.

In addition to the data referring to children with SNHL, this study also analyzed five children using cochlear implants, and all of them presented dysfunctions in the vestibular system and postural instabilities in the stabilometric evaluation. These findings corroborate those of other studies, which found vestibular dysfunctions [105,106,107,108,109,110] and balance changes [111,112,113,114,115] in their samples, composed of children using cochlear implants. Despite these findings, after cochlear implant surgery, the children have demonstrated satisfactory results in relation to speech and hearing [116,117,118,119,120,121], and as balance is a motor skill capable of being improved through rehabilitation exercises, children can benefit from vestibular rehabilitation and balance programs specific to the population, if referred in a timely manner.

Therefore, both children with hearing loss and associated vestibular dysfunction, as well as those undergoing cochlear implant surgery, should be evaluated and monitored by physiotherapists, in relation to their motor function and should be included in vestibular and balance rehabilitation programs to improve/adapt their development and motor performance.

Vestibular rehabilitation exercises have demonstrated satisfactory results in improving balance and reducing vestibular symptoms in children [122,123,124], being considered as key exercises to improve postural stability in children with vestibular disorders [124]. Therapeutic balance exercises have also demonstrated satisfactory results in improving postural stability and gait in children with SNHL and should be included in cases of vestibular hypofunction, whether through exercises, sports, and recreational practices, or virtual reality-based games [125,126,127,128].

Therefore, it is important that pediatric otorhinolaryngologists and audiologists observe not only the auditory aspects and speech of children with SNHL, but also motor function, and refer children with greater degrees of hearing loss and vestibular dysfunction for physiotherapeutic evaluation and motor rehabilitation, if necessary.

Furthermore, it is important that pediatric otorhinolaryngologist surgeons refer children undergoing cochlear implant surgery, who have developed imbalances and vestibular symptoms, for evaluation by a pediatric physiotherapist, as this professional works in the prevention and rehabilitation of motor development disorders, and rehabilitation vestibular and body balance, and would be able to accompany these children in the pre- and post-surgical period, demonstrating the importance of a physiotherapist in the interdisciplinary team for the motor rehabilitation of children with SNHL.

## 5. Conclusions

The children with SNHL presented more postural instabilities than those with normal hearing. Those with severe and profound hearing loss demonstrated the greatest instabilities in relation to the degrees of hearing loss, and children with hearing loss and associated vestibular dysfunction presented the greatest postural instabilities with regard to vestibular function. All children with cochlear implants had vestibular dysfunction and postural instabilities.

## Figures and Tables

**Table 1:** Characterization of the samples.

	Children with Normal Hearing		Children with Hearing Loss		*p*-Value
	*n*	(%)	*n*	(%)	
**Volunteers**	65	(100)	65	(100)	
**Sexes:**					
- Female	35	(53.8)	35	(53.8)	
- Male	30	(46.2)	30	(46.2)	
**Age, Years (Mean)**	9.0 ± 1.45		9.0 ± 1.45		1.000 ^a^
**Height**	1.34 ± 0.08		1.35 ± 0.09		0.573 ^a^
**Weight**	31.9 ± 8.71		31.7 ± 9.04		0.601 ^a^
**Body Mass Index (BMI)**	17.2 ± 3.27		16.8 ± 3.26		0.903 ^a^
**Z-Score (BMI)**	0.85 ± 1.24		0.53 ± 2.46		0.354 ^a^
**Handedness:**					
- Right-Handed	62	(95.4)	60	(92.3)	0.359 ^b^
- Left-Handed	3	(4.6)	5	(7.7)	
**Degrees of Hearing Loss:**					
- Mild and Moderate	--	--	17	(26.2)	
- Severe and Profound	--	--	48	(73.8)	
**Vestibular Dysfunction**	--	--	27	(41.5)	
**Hearing AIDS Users**	--	--	2	(3.1)	
**Cochlear Implant Users**	--	--	5	(7.7)	
**Etiology of Hearing Loss:**					
- Unknown	--	--	23	(35.4)	
- Rubella	--	--	18	(27.7)	
- Prematurity	--	--	7	(10.8)	
- Consanguinity of Parents	--	--	6	(9.2)	
- Use of Ototoxic Drugs	--	--	5	(7.7)	
- Hypoxia Peri or Postnatal	--	--	5	(7.7)	
- Meningitis Postnatal	--	--	1	(1.5)	

^a^ Student’s *t*-test; ^b^ Pearson’s Chi-squared test; AIDS: Amplifier individual device sonorous.

**Table 2:** Postural sway velocity of the center of pressure in antero-posterior and medio-lateral directions of children with normal hearing and sensorineural hearing loss (cm/s).

		Antero-Posterior				Medio-Lateral			
		Normal Hearing (*n* = 65)	Hearing Loss (*n* = 60)			Normal Hearing (*n* = 65)	Hearing Loss (*n* = 60)		
		Mean ± SD	Mean ± SD	DM (CI)	*p*-Value	Mean ± SD	Mean ± SD	DM (CI)	*p*-Value
	**Parallel feet**	1.56 ± 0.55	4.95 ± 2.42	−3.39 (−4.00 to −2.77)	0.001 ^a^	1.45 ± 0.52	1.97 ± 1.09	−0.52 (−0.81 to −0.22)	0.001 ^a^
**Eyes Open**	**Tandem feet**	2.84 ± 1.14	4.94 ± 3.12	−2.10 (−2.91 to −1.28)	0.000 ^a^	2.09 ± 0.62	3.30 ± 1.84	−1.21 (−1.68 to −0.73)	0.000 ^a^
	**One foot**	3.87 ± 1.43	5.29 ± 2.97	−1.42 (−2.23 to −0.60)	0.003 ^a^	3.49 ± 1.01	4.83 ± 2.52	−1.34 (−2.01 to −0.66)	0.001 ^a^
	**Parallel feet**	2.00 ± 0.75	2.91 ± 3.28	−0.91 (−1.73 to 0.08)	0.050 ^a^	1.83 ± 0.63	2.43 ± 1.65	−0.60 (−1.03 to −0.16)	0.002 ^a^
**Eyes Closed**	**Tandem feet**	4.61 ± 2.21	5.84 ± 2.83	−1.23 (−2.12 to −0.33)	0.005 ^a^	3.71 ± 1.51	4.90 ± 2.28	−1.19 (−1.86 to −0.51)	0.000 ^a^
	**One foot**	8.56 ± 4.21	8.58 ± 3.91	−0.02 (−1.46 to 1.42)	0.994 ^a^	6.93 ± 2.23	8.18 ± 3.69	−1.25 (−2.32 to −0.18)	0.246 ^a^

SD: Standard Deviation; DM: Difference of Means; CI: Confidence Interval; ^a^: Mann–Whitney test (with Holm–Bonferroni correction).

**Table 3:** Postural sway velocity of the center of pressure in antero-posterior direction of children with normal hearing and sensorineural hearing loss, according to degrees of hearing loss (cm/s).

		Normal Hearing (*n* = 65)	SNHL (MM) (*n* = 12)	SNHL (SP) (*n* = 26)	NH versus SNHL-MM			NH versus SNHL-SP			SNHL: MM versus SP		
		Mean ± SD	Mean ± SD	Mean ± SD	DM	CI 95%	*p*-Value	DM	CI 95%	*p*-Value	DM	CI 95%	*p*-Value
	Parallel feet	1.56 ± 0.55	1.85 ± 0.74	2.73 ± 2.24	−0.29	−0.65 to 0.07	0.308 ^a^	−1.17	−1.75 to −0.58	0.001 ^a^	−0.88	−2.23 to 0.47	0.218 ^a^
Eyes Open	Tandem feet	2.84 ± 1.14	3.52 ± 1.72	4.75 ± 2.91	−0.68	−1.45 to 0.09	0.241 ^a^	−1.91	−2.74 to −1.07	0.002 ^a^	−1.23	−3.07 to 0.61	0.218 ^a^
	One foot	3.87 ± 1.43	4.23 ± 1.87	5.74 ± 3.35	−0.36	−1.30 to 0.58	0.668 ^a^	−1.87	−2.86 to −0.87	0.003 ^a^	−1.50	−3.61 to 0.59	0.146 ^a^
	Parallel feet	2.00 ± 0.75	2.23 ± 0.70	2.25 ± 0.98	−0.23	−0.69 to 0.23	0.211 ^a^	−0.25	−0.62 to 0.12	0.416 ^a^	−0.02	−0.65 to 0.61	0.588 ^a^
Eyes Closed	Tandem feet	4.61 ± 2.21	5.63 ± 3.78	5.59 ± 1.90	−1.02	−2.58 to 0.54	0.365 ^a^	−0.98	−1.96 to 0.09	0.011 ^a^	0.04	−1.81 to 1.89	0.429 ^a^
	One foot	8.56 ± 4.21	8.26 ± 4.12	8.01 ± 3.47	0.30	−2.32 to 2.92	0.705 ^a^	0.55	−1.30 to 2.40	0.513 ^a^	0.25	−2.35 to 2.85	0.745 ^a^

SD: Standard Deviation; DM: Difference of Means; CI: Confidence Interval; MM: Mild and moderate sensorineural hearing loss; SP: Severe and profound sensorineural hearing loss. ^a^: Mann–Whitney test (with Holm–Bonferroni correction).

**Table 4:** Postural sway velocity of the center of pressure in medio-lateral direction of children with normal hearing and sensorineural hearing loss, according to degrees of hearing loss (cm/s).

		Normal Hearing (*n* = 65)	SNHL (MM) (*n* = 12)	SNHL (SP) (*n* = 26)	NH versus SNHL-MM			NH versus SNHL-SP			SNHL: MM versus SP		
		Mean ± SD	Mean ± SD	Mean ± SD	DM	CI 95%	*p*-Value	DM	CI 95%	*p*-Value	DM	CI 95%	*p*-Value
	**Parallel feet**	1.45 ± 0.52	1.62 ± 0.63	2.04 ± 0.77	−0.17	−0.50 to 0.16	0.474 ^a^	−0.59	−0.86 to −0.31	0.000 ^a^	−0.42	−0.93 to 0.09	0.129 ^a^
**Eyes Open**	**Tandem feet**	2.09 ± 0.62	2.81 ± 1.22	3.00 ± 1.26	−0.72	−1.18 to −0.25	0.074 ^a^	−0.91	−1.30 to −0.51	0.001 ^a^	−0.19	−1.07 to 0.69	0.631 ^a^
	**One foot**	3.49 ± 1.01	3.97 ± 1.39	4.94 ± 2.12	−0.48	−1.15 to 0.19	0.273 ^a^	−1.45	−2.10 to −0.79	0.001 ^a^	−0.97	−2.33 to 0.39	0.174 ^a^
	**Parallel feet**	1.83 ± 0.63	2.07 ± 0.70	2.12 ± 0.78	−0.24	−0.64 to 0.16	0.088 ^a^	−0.29	−0.60 to 0.02	0.043 ^a^	−0.05	−0.58 to 0.48	0.816 ^a^
**Eyes Closed**	**Tandem feet**	3.71 ± 1.51	4.51 ± 1.85	4.71 ± 1.28	−0.80	−1.77 to 0.17	0.070 ^a^	−1.00	−1.66 to −0.33	0.001 ^a^	−0.20	−1.24 to 0.84	0.297 ^a^
	**One foot**	6.93 ± 2.23	7.55 ± 2.41	7.90 ± 3.57	−0.62	−2.03 to 0.79	0.565 ^a^	−0.97	−2.20 to 0.26	0.809 ^a^	−0.35	−2.65 to 1.95	0.914 ^a^

SD: Standard Deviation; DM: Difference of Means; CI: Confidence Interval; MM: Mild and moderate sensorineural hearing loss; SP: Severe and profound sensorineural hearing loss. ^a^: Mann–Whitney test (with Holm–Bonferroni correction).

**Table 5:** Postural sway velocity of the center of pressure in antero-posterior direction of children with normal hearing and sensorineural hearing loss, according to vestibular function (cm/s).

		Normal Hearing (*n* = 65)	Hearing Loss (NVF) (*n* = 38)	Hearing Loss (VD) (*n* = 22)	NH versus SNHL-NVF			NH versus SNHL-VD			SNHL: NVF versus VD		
		Mean ± SD	Mean ± SD	Mean ± SD	DM	CI 95%	*p*-Value	DM	CI 95%	*p*-Value	DM	CI 95%	*p*-Value
	**Parallel feet**	1.56 ± 0.55	2.45 ± 1.93	9.26 ± 6.58	−0.89	−1.39 to −0.38	0.001 ^a^	−7.70	−9.32 to −6.07	0.069 ^a^	−6.81	−9.08 to −4.53	0.824 ^a^
**Eyes Open**	**Tandem feet**	2.84 ± 1.14	4.36 ± 2.63	5.94 ± 3.67	−1.52	−2.26 to −0.77	0.003 ^a^	−3.10	−4.11 to −2.08	0.001 ^a^	−1.58	−3.21 to 0.05	0.109 ^a^
	**One foot**	3.87 ± 1.43	5.26 ± 3.02	5.33 ± 2.97	−1.39	−2.26 to −0.51	0.011 ^a^	−1.46	−2.40 to −0.51	0.029 ^a^	−0.07	−1.67 to 1.53	0.860 ^a^
	**Parallel feet**	2.00 ± 0.75	2.23 ± 0.89	4.10 ± 5.15	−0.23	−0.55 to 0.09	0.215 ^a^	−2.10	−3.39 to −0.80	0.036 ^a^	−1.87	−3.57 to −0.16	0.311 ^a^
**Eyes Closed**	**Tandem feet**	4.61 ± 2.21	5.60 ± 2.58	6.26 ± 3.23	−0.99	−1.94 to −0.03	0.016 ^a^	−1.65	−2.87 to −0.42	0.033 ^a^	−0.66	−2.17 to 0.85	0.570 ^a^
	**One foot**	8.56 ± 4.21	8.09 ± 3.63	9.38 ± 4.31	0.47	1.15 to 2.09	0.488 ^a^	−0.82	−2.89 to 1.25	0.329 ^a^	−1.29	−3.37 to 0.79	0.073 ^a^

SD: Standard Deviation; DM: Difference of Means; CI: Confidence Interval; NVF: Children with SNHL and normal vestibular function; VD: Children with SNHL and vestibular dysfunction; ^a^: Mann–Whitney test (with Holm–Bonferroni correction).

**Table 6:** Postural sway velocity of the center of pressure in medio-lateral direction of children with normal hearing and sensorineural hearing loss, according to vestibular function (cm/s).

		Normal Hearing (*n* = 65)	Hearing Loss (NVF) (*n* = 38)	Hearing Loss (VD) (*n* = 22)	NH versus SNHL-NVF			NH versus SNHL-VD			SNHL: NVF versus VD		
		Mean ± SD	Mean ± SD	Mean ± SD	DM	CI 95%	*p*-Value	DM	CI 95%	*p*-Value	DM	CI 95%	*p*-Value
	**Parallel feet**	1.45 ± 0.52	1.91 ± 0.75	2.08 ± 1.52	−0.46	−0.70 to −0.21	0.001 ^a^	−0.63	−1.06 to −0.19	0.068 ^a^	−0.17	−0.75 to 0.41	0.485 ^a^
**Eyes Open**	**Tandem feet**	2.09 ± 0.62	2.94 ± 1.24	3.93 ± 2.49	−0.85	−1.21 to −0.48	0.001 ^a^	−1.84	−2.50 to −1.17	0.000 ^a^	−0.99	−1.95 to −0.02	0.187 ^a^
	**One foot**	3.49 ± 1.01	4.63 ± 1.96	5.17 ± 3.29	−1.14	−1.72 to −0.55	0.001 ^a^	−1.68	−2.58 to −0.77	0.056 ^a^	−0.54	−1.89 to 0.81	0.957 ^a^
	**Parallel feet**	1.83 ± 0.63	2.11 ± 0.75	3.00 ± 2.48	−0.28	−0.55 to −0.06	0.016 ^a^	−1.17	−1.83 to −0.50	0.008 ^a^	−0.89	−1.75 to −0.02	0.165 ^a^
**Eyes Closed**	**Tandem feet**	3.71 ± 1.51	4.65 ± 1.46	5.34 ± 3.25	−0.94	−1.54 to −0.33	0.000 ^a^	−1.63	−2.65 to −0.60	0.009 ^a^	−0.69	−1.91 to 0.53	0.602 ^a^
	**One foot**	6.93 ± 2.23	7.79 ± 3.22	8.86 ± 4.39	−0.86	−1.92 to 0.20	0.640 ^a^	−1.93	−3.36 to −0.49	0.105 ^a^	−1.07	−3.04 to 0.90	0.315 ^a^

SD: Standard Deviation; DM: Difference of Means; CI: Confidence Interval; NVF: Children with SNHL and normal vestibular function; VD: Children with SNHL and vestibular dysfunction; ^a^: Mann–Whitney test (with Holm–Bonferroni correction).

**Table 7:** Postural sway velocity of the center of pressure in antero-posterior and medio-lateral directions of children with cochlear implants (cm/s).

		Antero-Posterior (*n* = 5)	Medio-Lateral (*n* = 5)
		Mean ± SD	Mean ± SD
	Parallel feet	4.60 ± 2.18	2.56 ± 0.44
Eyes Open	Tandem feet	5.96 ± 1.31	4.13 ± 1.11
	One foot	5.77 ± 0.98	6.50 ± 2.09
	Parallel feet	2.27 ± 0.29	2.03 ± 0.16
Eyes Closed	Tandem feet	6.57 ± 1.17	4.66 ± 0.34
	One foot	7.41 ± 1.29	8.51 ± 2.40

SD: Standard Deviation.

## Data Availability

Not available.
